# Theoretical exploration of As-based mixed halide double perovskites A_3_AsI_6_ (A = K, Rb, and Cs) for photovoltaics applications using a DFT approach

**DOI:** 10.1039/d5ra06050h

**Published:** 2026-03-19

**Authors:** Muhammad Yar Khan, S. S. A. Shah, It Ee Lee, Qamar Wali, Tariq Usman, Yang Mu, Azim Khan, Abdullah Al Souwaileh

**Affiliations:** a Department of Physics, Qilu Institute of Technology Jinan 250200 Shandong P.R. China hmyarkhan@yahoo.com; b School of Mechanical and Electrical Engineering, Quanzhou University of Information Engineering Quanzhou 362000 China; c Faculty of Artificial Intelligence and Engineering, Multimedia University 63100 Cyberjaya Malaysia; d Centre for Smart Systems and Automation, Multimedia University 63100 Cyberjaya Malaysia; e Yangtze Delta Region Institute (Huzhou), University of Electronic Science and Technology of China Huzhou 313001 China; f Department of Chemistry, College of Science, King Saud University Riyadh 11451 Saudi Arabia

## Abstract

In this study, we explore the structural, electronic, optical, and elastic features of environmentally friendly lead-free mixed-halide double perovskites with the general composition A_3_AsI_6_ (A = K, Rb, and Cs), which are comprehensively analyzed using density functional theory (DFT). Our calculations reveal that the optimized lattice constants increase from 12.42 Å for K_3_AsI_6_ to 12.99 Å for Cs_3_AsI_6_, which is consistent with the progressive enlargement of the alkali metal ionic radii. To evaluate the electronic band structures, the Tran–Blaha-modified Becke–Johnson (TB-mBJ) potential was applied, with and without incorporating spin–orbit coupling (SOC), to achieve reliable estimations of the band gaps. The results reveal a consistent trend of decreasing band gap energies: 2.763 eV (mBJ) and 2.566 eV (mBJ + SOC) for K_3_AsI_6_ (indirect), 2.821 eV (mBJ) and 2.607 eV (mBJ + SOC) for Rb_3_AsI_6_, and 2.829 eV (mBJ) and 2.621 eV (mBJ + SOC) for Cs_3_AsI_6_. The density of states analyses further clarify the orbital contributions to the occupied and unoccupied bands. Elastic constants (*C*_ij_) confirm the mechanical stability of the materials, while Poisson's and Pugh's ratios indicate brittle behavior. Moreover, the calculated Debye temperatures suggest that K_3_AsI_6_ could better withstand thermal stresses induced by lattice vibrations than its Rb and Cs analogues. The optical characteristics, such as the dielectric function *ε*(*ω*), absorption coefficient *α*(*ω*), reflectivity *R*(*ω*), and refractive index *n*(*ω*), were comprehensively examined, revealing robust interactions with incident electromagnetic radiation. These comprehensive results underscore the potential of A_3_AsI_6_ (A = K, Rb, and Cs) double perovskites as viable candidates for next-generation optoelectronic applications, particularly in environmentally benign, lead-free technologies.

## Abstract 2

Density functional theory (DFT) is used to study the structural, electronic, optical, and photovoltaic properties of As-based double perovskites A_3_AsI_6_ (A = K, Rb, Cs), identifying promising candidates for high-efficiency solar cells.

## Graphical abstract

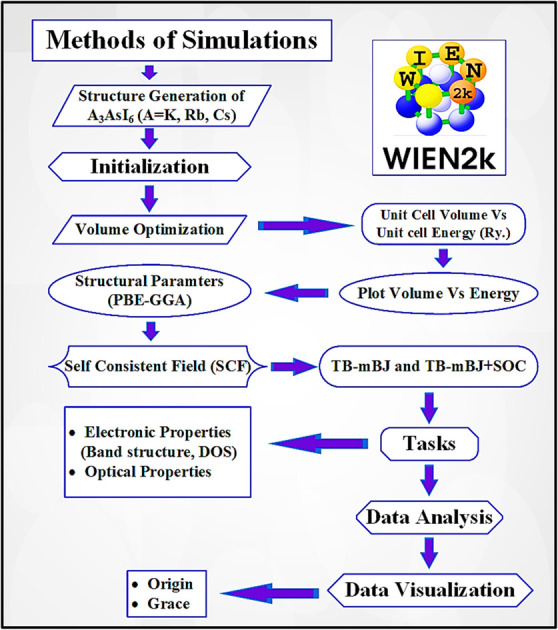

## 1 Introduction

With the rapid progress of industrialization, modern society faces pressing challenges arising from the depletion of fossil fuels and the escalation of environmental pollution. The extensive use of conventional energy sources, particularly in transportation and other high-energy-demand sectors, continues to drive greenhouse gas emissions, highlighting the urgent need for their clean, sustainable, and efficient energy alternatives.

In this regard, perovskite-derived materials have attracted research attention as viable candidates owing to their distinctive crystal structures and exceptional multifunctional properties. Their strong optical absorption, efficient charge transport, and tunable electronic features make them highly appropriate for next-generation energy conversion and optoelectronic applications, including solar cells, light-emitting diodes (LEDs), photodetectors, and radiation sensors. The general perovskite structure (ABX_3_) offers remarkable compositional flexibility, allowing for the precise optimization of physical and electronic properties. Within this class, halide-based perovskites, characterized by the presence of a halogen element at the X-site, stand out as notable compounds and have attracted particular research interest due to their compliant interaction and unresolved optoelectronic recital, positioning them at the forefront of sustainable energy research.^1,2^

Halide perovskites have gained significant attention in recent years because of their excellent optoelectronic properties, including tunable bandgaps, strong light absorption, high charge mobility, and remarkable power conversion efficiency. These attributes make them highly suitable for use in solar cells, LEDs, photodetectors, and other light-harvesting and optical devices. Moreover, wide-bandgap halide perovskites have shown great potential in advanced applications, such as lasers, scintillators, X-ray detectors, and high-frequency gas sensors. However, the widespread use of lead-based halide perovskites raises serious environmental and health concerns due to lead toxicity and the partial inherent stability of these materials.^3–5^ To address this issue, researchers have focused significant efforts on developing lead-free alternatives that retain the desirable optoelectronic properties of conventional perovskites while eliminating the associated environmental and health risks.^6–8^ Single and double halide perovskites have emerged as highly promising materials for efficient and stable optoelectronic applications. Among them, A_2_BX_6_-type double perovskites are particularly notable because they feature isolated [BX_6_] octahedra separated by alkali metal cations, reducing the three-dimensional connectivity compared with the conventional ABX_3_ structures. Advanced halide perovskite nanomaterials, such as quantum dots and nanocrystals, also show strong potential due to their quantum confinement, high defect tolerance, and excellent photoluminescence. These features make them suitable for applications in memory devices, bioimaging, and photocatalysis, highlighting their promise for next-generation optoelectronic technologies.^3,6,9^ The distinctive crystal framework of these compounds provides extensive compositional versatility, allowing fine adjustment of their thermodynamic stability and electronic characteristics through selective substitution at the A- and X-lattice sites. Experimental efforts focused on the synthesis and structural analysis of several A_3_BX_6_ perovskites have established crucial orientation facts for computational modeling. For example, Rb_3_InCl_6_ and Rb_3_InBr_6_ have been experimentally verified to adopt cubic *Fm*3̄*m* symmetry, characterized by discrete InX_6_ octahedral units and wide bandgaps greater than 4 eV, which underscores their promise for UV-transparent dielectric and deep-UV optoelectronic applications.^10,11^ Similarly, Cs_3_Bi_2_I_9_, despite having a slightly different A_3_B_2_X_9_ composition, exhibits structurally related zero-dimensional or layered motifs. Owing to its band gap of roughly 2 eV, it has emerged as a promising lead-free absorber material for solar energy conversion.^12–14^ One of the most advantageous features of the A_3_BX_6_ crystal framework lies in its ability to achieve band gap modulation through halide substitution. As the halogen element transitions from fluorine to iodine (F → Cl → Br → I), a progressive reduction in the band gap is observed. This trend primarily arises from the increase in ionic radii and the corresponding decrease in halogen electronegativity, which collectively contribute to the upward shift of the valence band maximum.^15–17^ Both experimental results and density functional theory (DFT) simulations confirm the same trend, offering valuable insights for the cogent scheme of materials optimized for assorted optoelectronic applications spanning electromagnetic F→ and Cl-based compositions act as effective photodetectors, whereas Br- and I-based counterparts show promising performance in visible-light photovoltaic and scintillation applications. Notwithstanding these auspicious features, the A_3_AsI_6_ (A = K, Rb, and Cs) family remains relatively unexplored. Introducing As(iii) at the B-site results in an *n*s^2^ lone-pair arrangement, which can induce local physical falsifications and defect-tolerant electronic position characteristics that could enhance optoelectronic performance.^18,19^ Initial computational studies indicate that A_3_AsI_6_ (A = K, Rb, and Cs) compounds have direct or approximately direct band gaps ranging from about 2.4 eV to 2.9 eV, revealing their potential applicability in photovoltaic and scintillation devices, governed by the exact nature of the band alignment.^20,21^ However, comprehensive first-principles investigations of their structural stability, electronic band structure, optical properties, and defect characteristics remain limited. These studies are critical for precisely gauging their latent potential as ecologically sociable, lead-free semiconducting materials for practical, expedient applications.

Jehangir *et al.* (2025) performed an extensive theoretical study on the structural, mechanical, electronic, optical, light-yield, and thermodynamic characteristics of eco-friendly, lead-free mixed-halide double perovskites with the general composition Rb_3_SbX_6_ (X = F, Cl, Br, and I). Their results indicate that the optimized lattice parameters systematically expand from 9.61 Å for Rb_3_SbF_6_ to 13.01 Å for Rb_3_SbI_6_, corresponding to the increasing ionic radii of the halogen atoms. The electronic band structures, evaluated using the Tran–Blaha modified Becke–Johnson (TB-mBJ) exchange–correlation functional, exhibited a consistent reduction in the band gap from 5.477 eV for the fluoride to 2.851 eV for the iodide member, confirming the expected trend along the halide series. The density of states (DOS) profiles offered a detailed understanding of the orbital interactions contributing to the valence and conduction regions. Optical characteristics, including the complex dielectric function *ε*(*ω*), absorption coefficient *α*(*ω*), reflectivity *R*(*ω*), and refractive index *n*(*ω*), revealed pronounced and tunable light–matter interactions across the series. In particular, the calculated ideal light yield for Rb_3_SbI_6_ underscores its suitability for scintillation-based applications. Furthermore, thermodynamic analyses involving temperature-dependent Gibbs free energy, unit cell volume, entropy (*S*, J mol^−1^ K^−1^), and bulk modulus (*B*, GPa) demonstrated negative Gibbs free energies, continuous volume expansion with temperature, increasing entropy, and a gradual decline in bulk modulus, collectively confirming robust thermal stability. These comprehensive findings position Rb_3_SbX_6_ (X = F, Cl, Br, and I) as promising candidates for radiation detection and advanced optoelectronic technologies.^22^

**Fig. 1:**
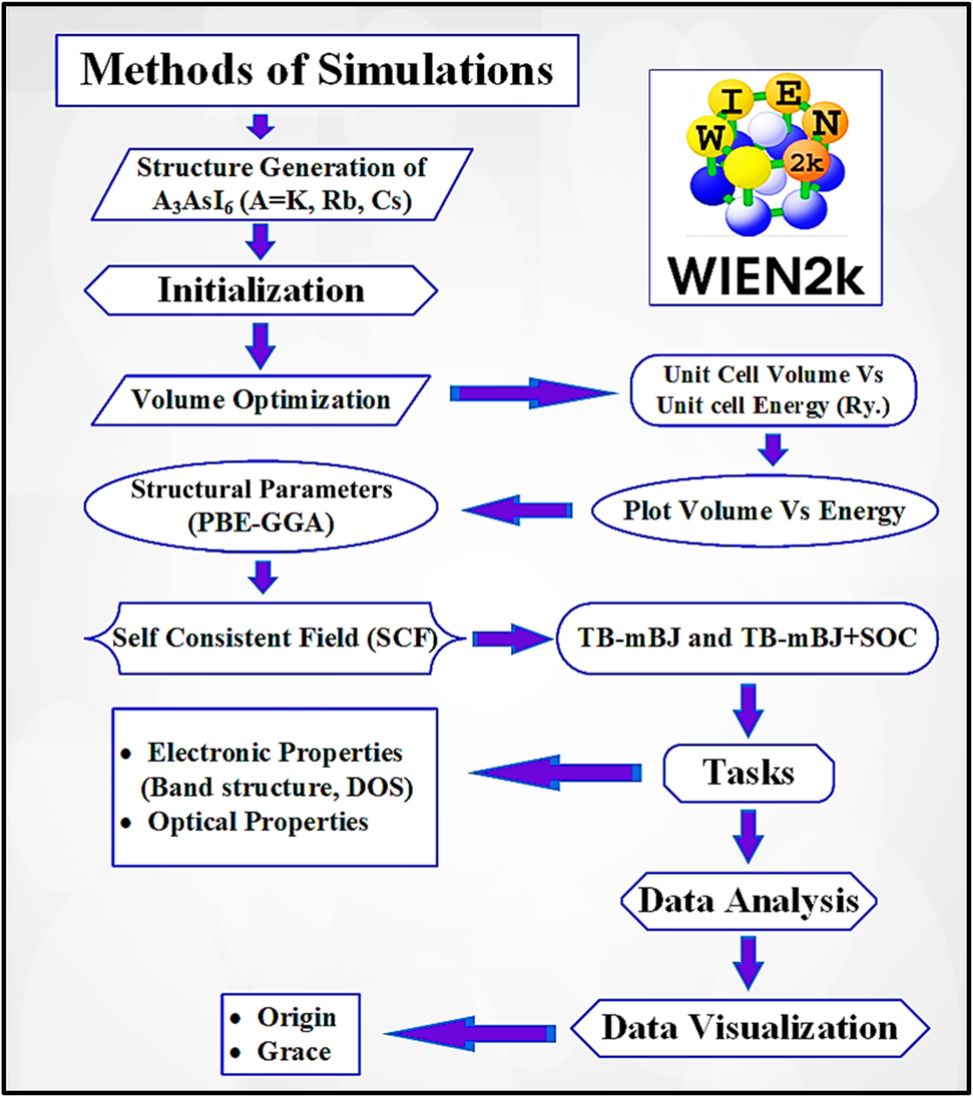
Method of simulations for the A_3_AsI_6_ (A = K, Rb, and Cs) compounds.

This study utilizes density functional theory (DFT) to comprehensively investigate the structural, electronic, elastic, thermodynamic, and optical characteristics of the A_3_AsI_6_ (A = K, Rb, and Cs) halide double perovskite family. Through an in-depth analysis of bandgap variations, dielectric behavior, and electronic transition mechanisms, this study aims to provide theoretical insights that can guide the development and optimization of environmentally benign, lead-free materials for advanced photovoltaic absorber applications (Fig. 1).

## 2 Method of simulations

The structural, electronic, and optical characteristics of the cubic halide perovskite were investigated using the full-potential linearized augmented plane wave (FP-LAPW) approach within the framework of density functional theory (DFT), as implemented in the WIEN2k software package. The exchange–correlation interactions were treated using the Perdew–Burke–Ernzerhof (PBE) functional within the generalized gradient approximation (GGA). The equilibrium lattice constants and cell volumes were obtained by fitting the calculated total energies to the Birch–Murnaghan equation of state.^23^ A 10 × 10 × 10 *k*-point grid was employed to ensure accurate Brillouin zone sampling during structural relaxation.1
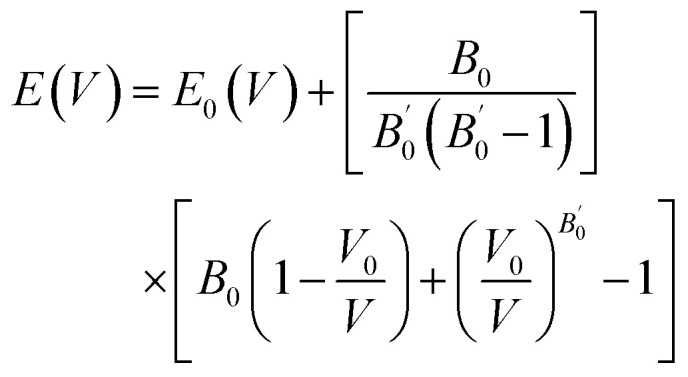


The condition for the plane-wave cutoff defined as *R*_MT_ × *K*_max_ = 8 was implemented, with the maximum angular momentum quantum number *l*_max_ set to 12, and *G*_max_ specified as 12. In the WIEN2k calculations, a core–valence separation was applied using a core-state cutoff energy of −6.0 Ry. Convergence in SCF calculations was achieved with thresholds of 0.0001 Ry for total energy and 0.0001 e for charge density.

Furthermore, the robustness of the designed compounds was analyzed by determining the Goldschmidt tolerance factor (*τ*_F_) and formation enthalpies (Δ*H*_F_):^24^2
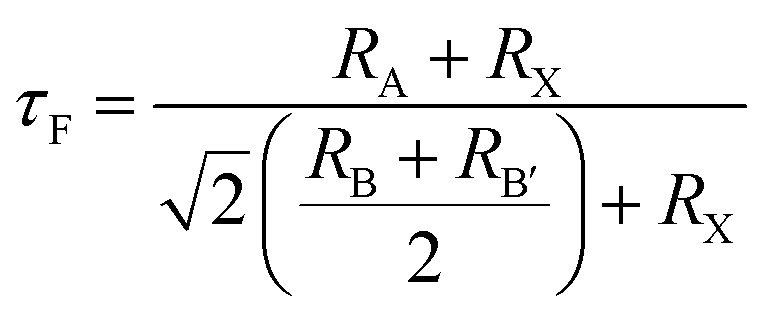
and

3 Δ*H*_F_ = *E*_(K/Rb/Cs)_3_AsI_6__ − 2*E*_K/Rb/Cs_ − *E*_As_ − 6*E*_I_.

The PBE-GGA functional was utilized to correctly capture the fundamental structural characteristics and ground-state configuration of the system while recognizing its inherent shortcomings in precisely determining optoelectronic properties. To address this limitation, the TB-mBJ potential, applied both with and without spin–orbit coupling, was adopted, demonstrating high reliability in yielding precise electronic band gap values.^25^ Moreover, an inclusive evaluation of the photosensitive characteristics was conducted to determine the aptness of these constituents for photonic and optoelectronic applications.^26^ An essential parameter for assessing a material's optical behavior is its complex dielectric function, comprising a real part *ε*_1_(*ω*) and an imaginary part *ε*_2_(*ω*). The real portion *ε*_1_(*ω*) determines the material's capacity to store electromagnetic energy and refract light, thereby affecting its reflectance and transmittance properties. In contrast, the imaginary portion *ε*_2_(*ω*) characterizes energy dissipation and absorption processes, establishing the material's captivation spectrum over different energy ranges. The relationship between *ε*_1_(*ω*) and *ε*_2_(*ω*) is rigorously described by the Kramers–Kronig relationships, as given in eqn (4) and (5).^27^4
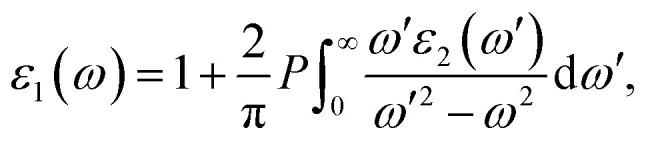
5
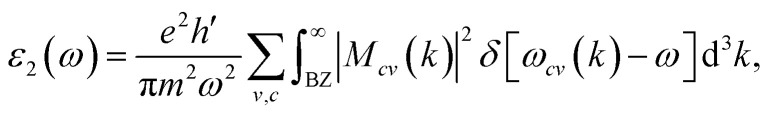
6
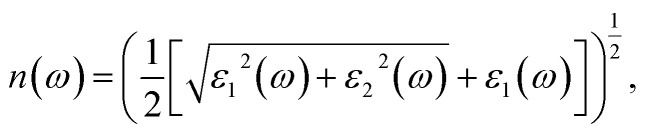
7
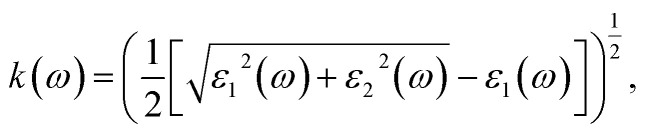
8
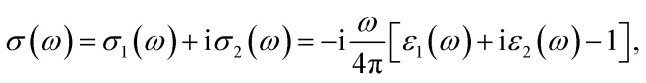
9
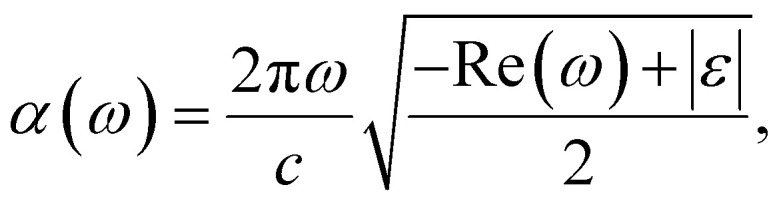
10
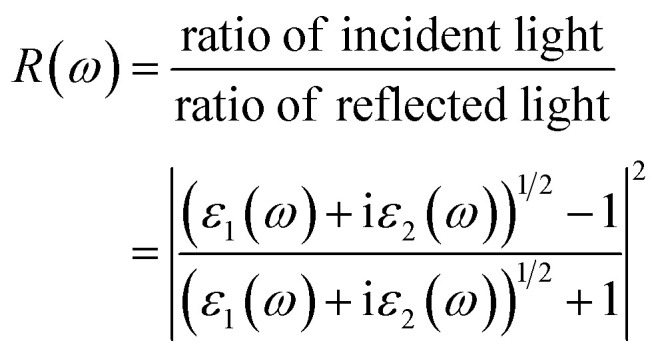
and11
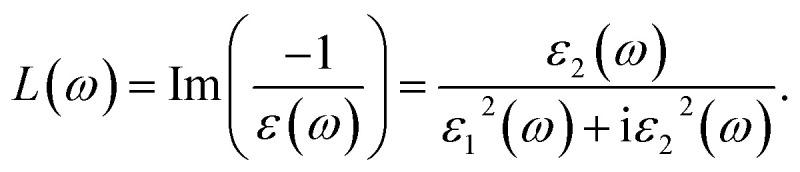


The wave vector *k* and principal quantum number *P* are defined as per standard conventions, with *h* representing Planck's constant, *h*(*ω*) denoting the angular frequency, and *M* denoting the molar mass. The components of the elastic tensor were evaluated following the methodology outlined by Morteza Jamal^28–30^ within the WIEN2k computational suite, allowing for the characterization of both spherical and planar harmonic vibrational modes in the studied material.

## 3 Results and discussion

### 3.1 Structural properties

**Fig. 2:**
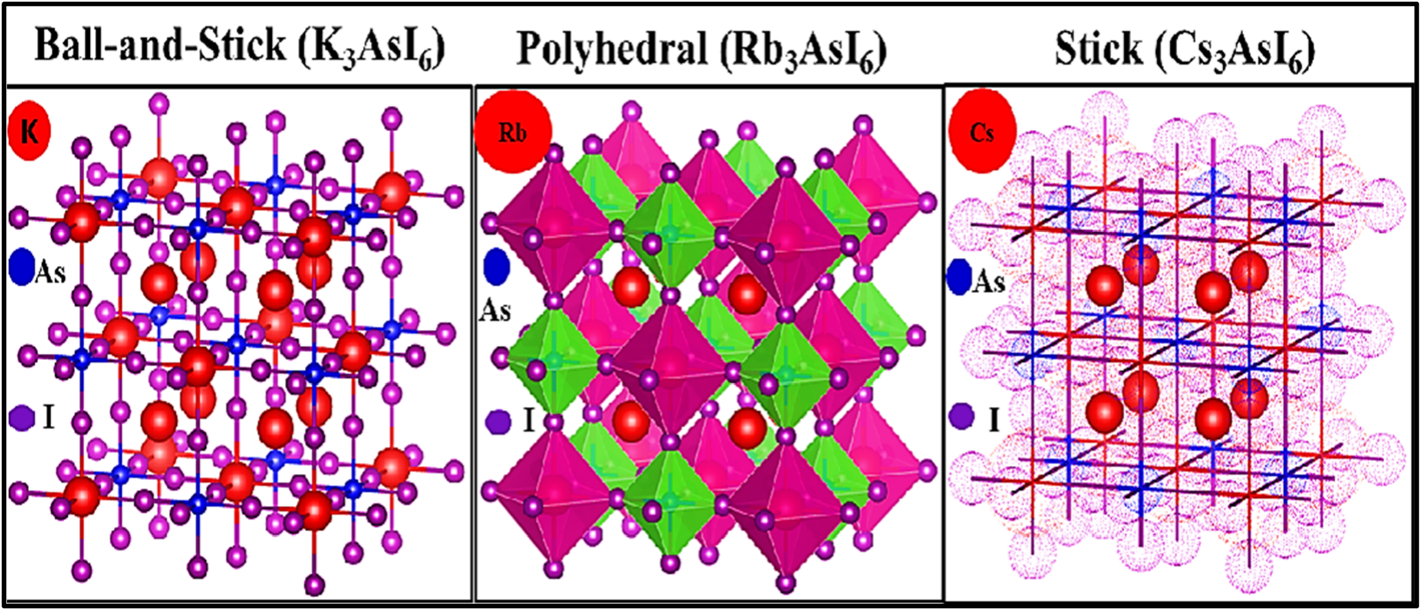
Crystal structures of the A_3_AsI_6_ (A = K, Rb, and Cs) halide DPs.

**Fig. 3:**
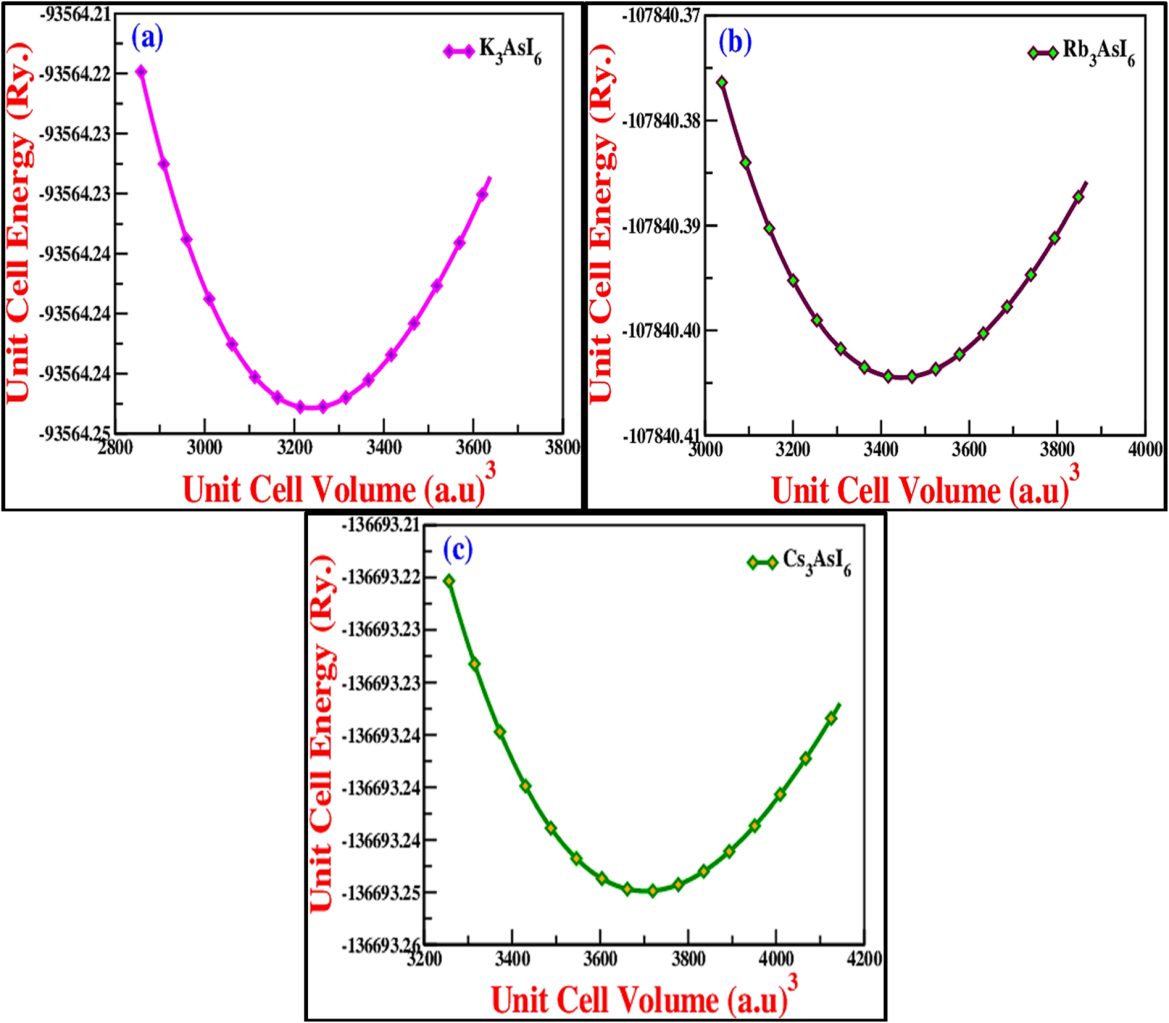
(a–c) Unit cell energy *vs.* unit cell volume plots for the A_3_AsI_6_ compounds where A = K, Rb, and Cs, respectively.

All computations were performed on the A_3_AsI_6_ (A = K, Rb, and Cs) halide compounds, which adopted a stable face-centered cubic configuration corresponding to the *Fm*3̄*m* (225) space group. Structural optimizations were performed until the systems reached their ground-state geometries, employing generalized gradient approximation (GGA) with the Perdew–Burke–Ernzerhof (PBE) functional. Volume optimization was carried out using the Birch–Murnaghan equation of state to determine the equilibrium lattice parameter *a*_0_[Å] and bulk modulus *B*_0_ (GPa), as shown in Table 1.^31^ A_3_AsI_6_ compounds (A = K, Rb, and Cs) espouse a face-centered cubic (FCC) crystal structure, with the A-site cations positioned at the Wyckoff coordinates (0.5, 0, 0), arsenic atoms occupying the epicenter of the unit cell at (0, 0, 0), and iodine atoms located at (0.23, 0, 0).^21,32^ The optimized crystal structures of the cubic (C-phase) A_3_AsI_6_ (A = K, Rb, and Cs) compounds are presented in Fig. 2, along with their corresponding energy–volume curves depicted in Fig. 3a–c. To thoroughly examine the structural properties of A_3_AsI_6_, key input structures, lattice constants, space group symmetries, and accurate atomic positions were employed.

**Table 1:** Structural properties of the A_3_AsI_6_ (A = K, Rb, and Cs) halide DPs

Materials	*a*_0_ [Å]	*V*_0_ [au]^3^	*B*_0_ [GPa]	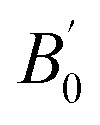	*E*_0_ [Ry.]	*τ*_F_	Δ*H*_F_ [eV per atom]	*ρ* [g cm^−3^]
K_3_AsI_6_	12.42	3237.51	14.48	5.00	−93564.24	0.93	−1.18	3.30
Rb_3_AsI_6_	12.68	3445.18	13.40	5.00	−107840.40	0.90	−1.16	3.55
Cs_3_AsI_6_	12.99	3703.01	12.52	5.00	−136693.24	0.94	−1.10	3.74
**Rb**_**3**_ **SbI**_**6**_	**13.01^a^**	**3717.55^a^**	**12.78^a^**	**5.00^a^**	**−116285.70^a^**	**0.94^a^**	**−1.34^a^**	**0.13^a^**

a Ref. 22.

Table 1 presents the improved physical structures along with the calculated solidity norms for all alignments. Besides the equipoise bulk modulus *B*_0_ (GPa), its pressure derivative 
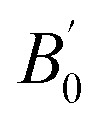
 serves as an important mechanical indicator, reflecting the material's confrontation with volume compression under external pressure. A gradual increase in lattice constants, coupled with a corresponding decrease in *B*_0_ (GPa), is observed as the cation is substituted from K to Cs. The bulk modulus *B*_0_ (GPa) reflects the resistance of the material to compression, where a smaller value signifies enhanced structural flexibility. Consequently, the calculated bulk moduli follow the trend *B*_0_(K_3_AsI_6_) > *B*_0_(Rb_3_AsI_6_) > *B*_0_(Cs_3_AsI_6_), indicating gradual lattice softening as the cation size increases.

Goldsmith's tolerance factor (*τ*_F_) serves as a key metric for assessing the structural stability of double perovskites. According to Goldsmith's criterion, a value approaching unity signifies an ideal and stable cubic architecture, while a tolerance factor ranging from 0.8 to 1.0 is generally regarded as necessary to stabilize the three-dimensional DP phases. As shown in Table 1, the calculated (*τ*_F_) values for the studied halide complexes fall within the defined stability window, suggesting their potential to retain a cubic phase. Additionally, to evaluate their thermodynamic favorability in the cubic conformation, the formation energies (Δ*H*_F_) of A_3_AsI_6_ (A = K, Rb, and Cs) were determined.^33,34^ The total energy of the A_3_AsI_6_ compounds is denoted as *E*(A_3_AsI_6_) while the reference energies of the constituent elements in their bulk forms are represented by *E*(K/Rb/Cs), *E*(As), and *E*(I). All the computed formation energies (Δ*H*_F_) are negative, indicating that these compounds are thermodynamically stable and may be feasible candidates for novel material synthesis.

### 3.2 Electronic properties

**Fig. 4:**
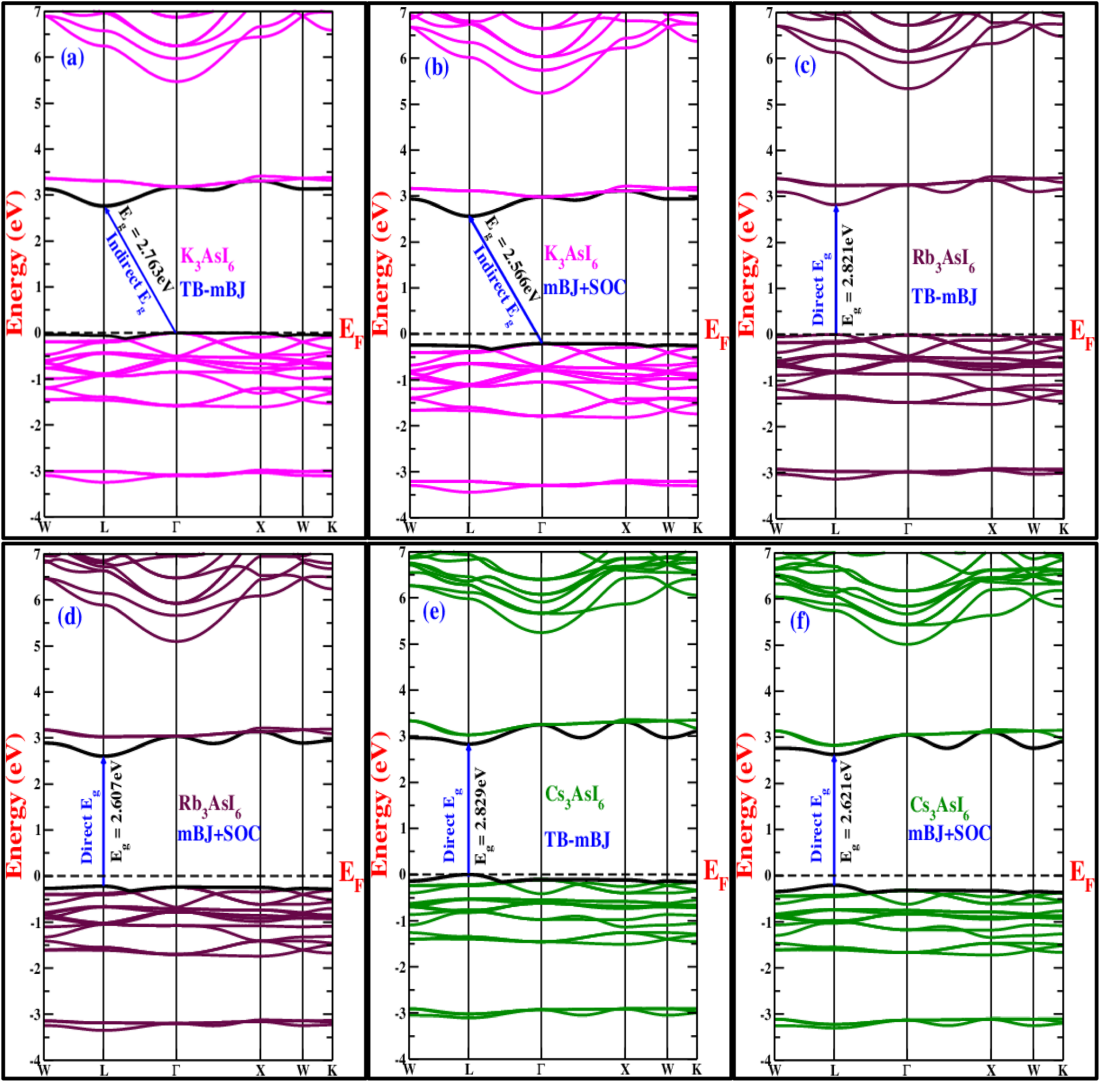
(a–f) Band structure plots of A_3_AsI_6_ (A = K, Rb, and Cs) with and without SOC.

To investigate the electronic properties of A_3_AsI_6_ (A = K, Rb, and Cs), band structure calculations were performed using the modified Becke–Johnson (TB-mBJ) exchange–correlation potential, both with and without spin–orbit coupling (SOC). The choice of exchange–correlation functional strongly affects the accuracy of the computed bandgap, making the use of TB-mBJ essential for reliable predictions, as standard PBE-GGA results can deviate from previously reported data. The electronic band structures and corresponding total density of states (TDOS) were computed along the principal high-symmetry directions of the Brillouin zone, as depicted in Fig. 4, with the Fermi level consistently set at 0 eV. The analysis indicates that the highest occupied valence band and the lowest unoccupied conduction band reside at different high-symmetry points, confirming the presence of both direct and indirect bandgaps. Using the TB-mBJ approach, with and without spin–orbit coupling (SOC), the bandgap energies were determined as follows: for K_3_AsI_6_, 2.763 eV (mBJ) and 2.566 eV (mBJ + SOC) indicate an indirect gap; for Rb_3_AsI_6_, 2.821 eV (mBJ) and 2.607 eV (mBJ + SOC) correspond to a direct L–L transition; and for Cs_3_AsI_6_, 2.829 eV (mBJ) and 2.621 eV (mBJ + SOC) show a direct L–L transition. These values are comparable with the reported 2.85 eV (TB-mBJ) for Rb_3_SbI_6_.^22^ These findings indicate that these materials can be categorized as wide-bandgap semiconductor devices. Owing to their comparatively large bandgap values, the halide double perovskites in this series are predicted to show reduced photon emission per absorbed photon, rendering them highly suitable for ultraviolet (UV) optoelectronic devices. Accordingly, these compounds possess significant potential for use in UV photodetectors, UV LEDs, scintillation sensors, and other advanced optoelectronic systems.^21,23^

A thorough analysis of the electronic band structure necessitates assessing how each atomic orbital contributes to the material's overall electronic states.

**Fig. 5:**
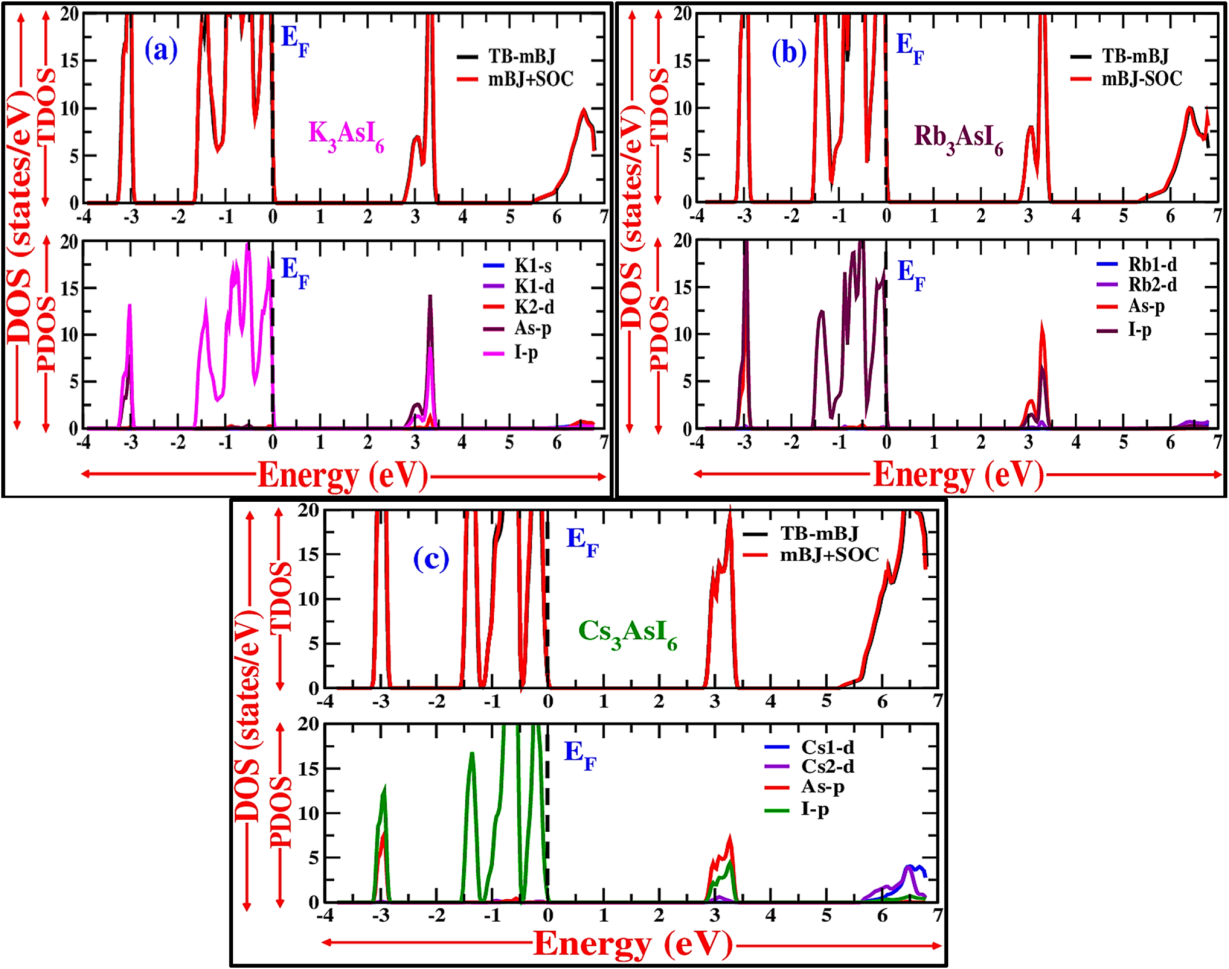
(a–c) DOS plots of the A_3_AsI_6_ compounds where A = K, Rb, and Cs, respectively, with and without SOC.

Fig. 5a–c presents the partial density of states (PDOS), which was evaluated and depicted over the energy range from −4 eV to +7 eV. For A_3_AsI_6_ (A = K, Rb, and Cs), the valence band extends from approximately −4 eV to 0 eV, with its maximum coinciding with the Fermi level. The relatively higher density of states within the valence band compared with that in the conduction band suggests a predominant p-type electronic behavior. The PDOS analysis shows that the valence band is mainly dominated by the p-orbitals of halide ions (I), while the contribution from arsenic p-orbitals is very small in the energy range from around −3.3 to −2.9 eV and −1.7 to 0 eV. In contrast, the conduction band is mainly formed by the p-orbitals of both As and I, particularly in the energy window between 2.9 and 3.5 eV for these materials. These findings confirm the semiconducting nature of A_3_AsI_6_ and underscore their potential for optoelectronic applications. Furthermore, the calculated density of states corroborates the incidence of band gaps that are unswerving with their semiconducting characteristics.^25^

Moreover, the effective mass plays a vital role in assessing the electronic and optical performance of materials, including their light response and electrical transport behavior. As the motion of charge carriers is inversely related to the effective masses of electrons and holes, larger effective mass values correspond to reduced carrier mobility.^35,36^Eqn (12) is used to determine the effective inertia by applying a non-linear fitting procedure to the electronic band structure data:12
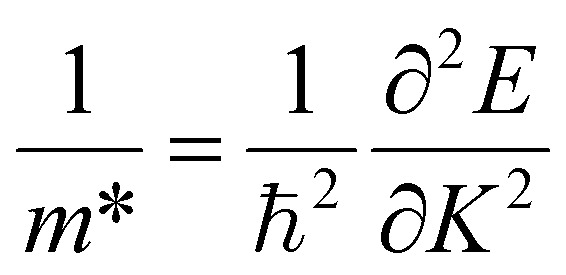


Additionally, the exciton binding energy (*E*^ex^_b_) was evaluated based on the effective masses of the charge carriers in conjunction with the static (zero-frequency) dielectric constant *ε*_1_(0), as given by eqn (13). This energy represents the amount required to separate an exciton, a Coulombically bound electron–hole pair, into free charge carriers. For photovoltaic applications, materials with lower exciton binding energies are desirable because they enable more efficient photon absorption and improve charge separation and collection efficiency.13
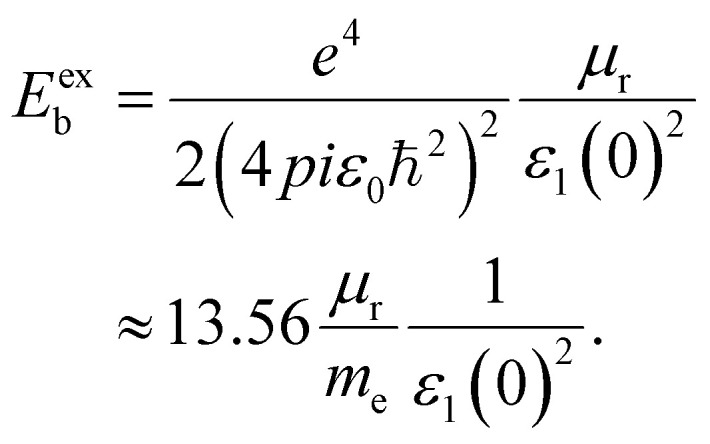


The effective masses and exciton binding energies for the A_3_AsI_6_ (A = K, Rb, and Cs) are illustrated in Table 2. Rb_3_SbI_6_ is found to exhibit higher electron and hole effective masses compared with K_3_SbI_6_ and Cs_3_SbI_6_, demonstrating reduced carrier mobility and improved scattering or localization effects.^36^ Besides, the calculated exciton binding energies (*E*^ex^_b_) for A_3_AsI_6_ (A = K, Rb, and Cs) are pointedly higher than those reported for Rb_2_CuAsF_6_ (0.29 eV)^37^ and In_2_AgSbCl_6_ (0.058 eV).^38^ These preeminent values designate strong Coulomb interactions amid electrons and holes, emphasizing the possibility of A_3_AsI_6_ (A = K, Rb, and Cs) compounds as effective absorber materials in photovoltaic applications.

**Table 2:** Calculated effective masses and exciton binding energies of the A_3_AsI_6_ (A = K, Rb, and Cs) compounds

Parameters	Method	K_3_AsI_6_	Rb_3_AsI_6_	Cs_3_AsI_6_
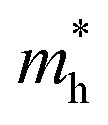	TB-mBJ (SOC)	0.447 (0.318)	0.077 (0.076)	0.076 (0.060)
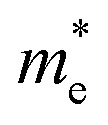	TB-mBJ (SOC)	0.040 (0.042)	0.043 (0.042)	0.037 (0.041)
*E* ^ex^_b_	TB-mBJ (SOC)	0.829 (0.808)	0.618 (0.622)	0.666 (0.585)

### 3.3 Optical properties

**Fig. 6:**
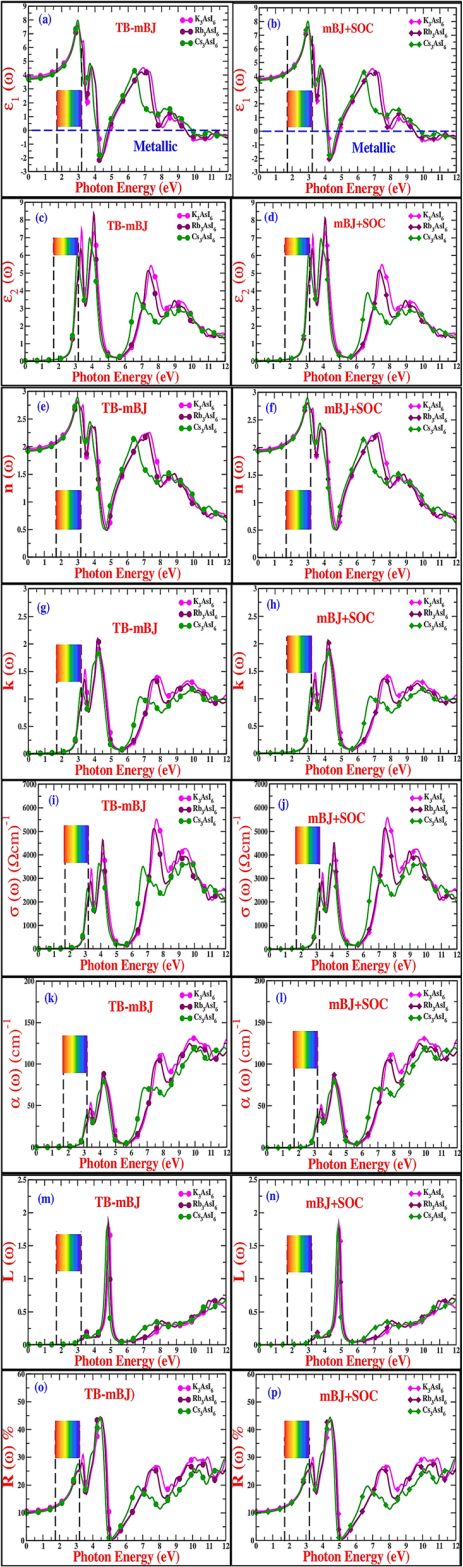
(a–p) Optical plots of the A_3_AsI_6_ (A = K, Rb, and Cs) compounds with and without SOC.

To better understand the light-interaction behavior of the halide double perovskites A_3_AsI_6_ (A = K, Rb, and Cs), both the real and imaginary parts of their dielectric functions were thoroughly examined, revealing their promising capability as efficient light-harvesting materials for clean electricity generation, as illustrated in Fig. 6a and b. The computed dielectric spectra span a photon energy range of 0 to 12 eV. The values of the static dielectric constant, *ε*(0), were determined to be 3.872 (mBJ) and 3.850 (mBJ + SOC) for K_3_AsI_6_, 3.749 (mBJ) and 3.746 (mBJ + SOC) for Rb_3_AsI_6_, and 3.700 (mBJ) and 3.708 (mBJ + SOC) for Cs_3_AsI_6_, in comparison with Rb_3_SbI_6_, whose value is 3.40 (TB-mBJ).^22^ Elsewhere, at the static boundary, all conformations display increasing dielectric values, reaching prominent crests of 7.740 at 2.95 eV (mBJ) and 7.641 at 2.97 eV (mBJ + SOC) for K_3_AsI_6_, 7.571 at 2.97 eV (mBJ) and 7.560 at 2.99 eV (mBJ + SOC) for Rb_3_AsI_6_, and 7.960 at 2.95 eV (mBJ) and 8.002 at 2.9 5 eV (mBJ + SOC) for Cs_3_AsI_6_, in comparison with Rb_3_SbI_6_ whose value is 6.35 at 2.97 eV (TB-mBJ),^22^ as depicted in Fig. 6a and b. In other regions, beyond the primary peak, the dielectric response exhibits additional enhancements, manifested as multiple smaller peaks originating from interband electronic transitions. The real part of the dielectric function becomes negative between 4 eV and 5 eV, reflecting metallic-like characteristics in this energy range, in accordance with Penn's model predictions. Fig. 6c and d illustrates the verge and top Photon energies corresponding to peaks in the imaginary part of the dielectric function, *ε*_2_(*ω*), for the A_3_AsI_6_ (A = K, Rb, and Cs) series. The threshold energies of *ε*_2_(*ω*) were found to be 2.23 eV (mBJ) and 2.07 eV (mBJ + SOC) for K_3_AsI_6_, 2.25 eV (mBJ) and 2.09 eV (mBJ + SOC) for Rb_3_AsI_6_, and 2.22 eV (mBJ) and 2.08 eV (mBJ + SOC) for Cs_3_AsI_6_, in comparison with Rb_3_SbI_6_ whose value is 2.67 eV (TB-mBJ). The corresponding maximum *ε*_2_(*ω*) values reached 7.485 at 3.36 eV (mBJ) and 7.117 at 3.36 eV (mBJ + SOC) for K_3_AsI_6_, 8.504 at 4.10 eV (mBJ) and 8.135 at 4.10 eV (mBJ + SOC) for Rb_3_AsI_6_, and 7.046 at 3.85 eV (mBJ) and 7.011 at 3.88 eV (mBJ + SOC) for Cs_3_AsI_6_, in comparison with Rb_3_SbI_6_ whose value is 7.50 at 4.50 eV (TB-mBJ).^22^ The intensity of these peaks increases at higher energies, which approach the near-visible (VIS) range, underscoring the correctness of these materials for optoelectronic applications within this spectral region.^21^

Fig. 6e and f depict the computed energy-dependent refractive index, *n*(*ω*), obtained using the mBJ method with and without SOC, highlighting its dependence on the group velocity, incident photon energy, and the inherent properties of the material. The refractive index offers critical evidence about the material's optical behavior by measuring the extent to which light is bent as it travels through the medium.^39,40^ The static refractive index values *n*(0) for A_3_AsI_6_ (A = K, Rb, and Cs) are found to be 1.968, 1.936, and 1.923 using mBJ, while 1.962, 1.935, and 1.925 using mBJ + SOC, in comparison with Rb_3_SbI_6_ whose value is 1.84 (TB-mBJ),^22^ respectively. A progressive increase in *n*(*ω*) is observed with increasing energy, reaching peak values of 2.834 at 2.97 eV (mBJ) and 2.840 at 2.98 eV (mBJ + SOC) for K_3_AsI_6_, 2.805 at 3.00 eV (mBJ) and 2.817 at 3.01 eV (mBJ + SOC) for Rb_3_AsI_6_, and 2.899 at 2.95 eV (mBJ) and 2.905 at 2.97 eV (mBJ + SOC) for Cs_3_AsI_6_, in comparison with Rb_3_SbI_6_ whose value is 2.39 at 4.24 eV (TB-mBJ). Fig. 6g and h shows the extinction coefficient *K*(*ω*), representing the imaginary part of the complex refractive index and indicating the extent of photon energy absorption within the solid. The onset of *K*(*ω*) is observed at 2.234 eV, 2.261 eV, and 2.218 eV using the mBJ approach, while it shifts to 2.105 eV, 2.143 eV, and 2.089 eV when incorporating mBJ + SOC for A3_33AsI6_66 (A = K, Rb, and Cs), in comparison with Rb_3_SbI_6_ whose value is 2.64 (TB-mBJ), respectively. The consistent topmost standards are 1.944 at 4.21 eV (mBJ) and 1.927 at 4.21 eV (mBJ + SOC) for K_3_AsI_6_, 2.126 at 4.13 eV (mBJ) and 2.073 at 4.18 eV (mBJ + SOC) for Rb_3_AsI_6_, and 1.851 at 4.26 eV (mBJ) and 1.874 at 4.10 eV (mBJ + SOC) for Cs_3_AsI_6_, further corroborating the ocular activity in the near-UV section, in comparison with Rb_3_SbI_6_ with a value of 1.23 at 3.41 eV (TB-mBJ).^22^ These findings align with the trends observed in the imaginary component of the dielectric function, *ε*_2_(*ω*), and the absorption coefficient, *α*(*ω*), revealing protuberant optical evolutions near the ultraviolet region. Reflectance, which is a material's aptitude to mirror incident electromagnetic energy, is a key factor in optoelectronic applications and the development of energy-efficient devices. Typically, metals show high reflectivity due to their abundance of free charge carriers, while semiconductors display comparatively lower values. The relatively low reflectivity measured for the A_3_AsI_6_ compounds (A = K, Rb, and Cs) corroborates their semiconducting nature.^21^

Optical conductivity *σ*(*ω*) serves as a measure of the charge-carrier dynamics induced by light. Fig. 6i and j depicts the simulated *σ*(*ω*) fields for Pb-free halide double perovskites A_3_AsI_6_ (A = K, Rb, and Cs). Moreover, *σ*(*ω*) remains negligible underneath the ocular band gap, with intended cutoff energies for A_3_AsI_6_ (A = K, Rb, and Cs) found to be 2.080 eV, 2.097 eV, and 2.071 eV using mBJ, while 2.088 eV, 2.102 eV, and 2.075 eV using mBJ + SOC, respectively. Extreme optical conductivities are observed at 5566.36 Ω^−1^ cm^−1^ at 7.55 eV (mBJ) and 5604.8 Ω^−1^ cm^−1^ at 7.54 eV (mBJ + SOC) for K_3_AsI_6_, 5157.52 Ω^−1^ cm^−1^ at 7.37 eV (mBJ) and 5167.95 Ω^−1^ cm^−1^ at 7.39 eV (mBJ + SOC) for Rb_3_AsI_6_, and 3563.07 Ω^−1^ cm^−1^ at 6.66 eV (mBJ) and 3652.62 Ω^−1^ cm^−1^ at 3.86 eV (mBJ + SOC) for Cs_3_AsI_6_, in comparison with Rb_3_SbI_6_, with a value of 4894.68 Ω^−1^ cm^−1^ (7.50 eV) (TB-mBJ).^22^ The absorption coefficient *α*(*ω*), depicted in Fig. 6k and l, quantifies the material's ability to diminish the intensity of incoming radiation.^29,41^ All complexes display noticeable absorption in the ultraviolet region, with prime peaks at 132.98 cm^−1^ at 9.89 eV (mBJ) and 131.78 cm^−1^ at 9.90 eV (mBJ + SOC) for K_3_AsI_6_, 125.94 cm^−1^ at 9.66 eV (mBJ) and 125.55 cm^−1^ at 9.59 eV (mBJ + SOC) for Rb_3_AsI_6_, and 120.46 cm^−1^ at 9.89 eV (mBJ) and 123.21 cm^−1^ at 10.01 eV (mBJ + SOC) for Cs_3_AsI_6_, in comparison with Rb_3_SbI_6_ whose value is 120.37 × 10^4^ cm^−1^ (9.63 eV) (TB-mBJ), confirming strong UV absorption performance pertinent for optoelectronic applications.^21^

Fig. 6m and n shows the premeditated energy loss, *L*(*ω*), for the A_3_AsI_6_ (A = K, Rb, and Cs) double perovskites. The findings reveal that energy loss remains low in the regions associated with the peak absorption coefficients, implying that *L*(*ω*) exerts only a minor influence on the overall optical behavior of these materials. The figured *L*(*ω*) peak standards are 1.796 at 4.92 eV (mBJ) and 1.718 at 4.93 eV (mBJ + SOC) for K_3_AsI_6_, 1.918 at 4.80 eV (mBJ) and 1.820 at 4.83 eV (mBJ + SOC) for Rb_3_AsI_6_, and 1.801 at 4.77 eV (mBJ) and 1.737 at 4.75 eV (mBJ + SOC) for Cs_3_AsI_6_, compared with Rb_3_SbI_6_ whose value is 1.70 at 5.13 eV (TB-mBJ),^22^ respectively. The relatively low peak powers imply that energy loss has a negligible effect on the absorption characteristics of the considered complexes. Overall, the ocular investigation demonstrates that these Pb-free halide DPs are auspicious materials for light-harvesting optoelectronic applications due to their sturdy absorption proficiencies, satisfactory scattering performance, small reflectivity in the visible region, and negligible energy loss observed in the near-ultraviolet region.^42^

Reflectivity, which represents the ratio between the intensities of incident and reflected radiation,^24,43^ is depicted in Fig. 6o and p. The *R*(*ω*) spectra exhibit lower reflectivity in the discernible section compared with the near-ultraviolet (UV) range. As shown in Fig. 6o and p, all investigated A_3_AsI_6_ (A = K, Rb, and Cs) compounds exhibit pronounced absorption in the ultraviolet region. The maximum reflectivity peaks are designed to be 41.826% at 4.59 eV (mBJ) and 41.475% at 4.56 eV (mBJ + SOC) for K_3_AsI_6_, 44.754% at 4.51 eV (mBJ) and 43.934% at 4.52 eV (mBJ + SOC) for Rb_3_AsI_6_, and 43.988% at 4.39 eV (mBJ) and 44.519% at 4.44 eV (mBJ + SOC) for Cs_3_AsI_6_, in comparison with Rb_3_SbI_6_ whose value is 39.44 at 4.91 eV (TB-mBJ). These results underscore the suitability of these materials for use in ultraviolet photodetectors, light-emitting diodes, photovoltaic devices, and other optoelectronic technologies. Moreover, improved ultraviolet irradiation leads to improved absorption; nevertheless, energy loss remains a critical factor because it establishes a channel for energy dissipation that may impair overall device efficiency.^21^

### 3.4 Mechanical properties

Employing computational models and nonlinear differential equations to study the mechanical behavior of materials. The evaluation follows Born's mechanical stability criteria C_11_–C_12_ > 0, C_44_ > 0, C_11_ + 2C_12_ > 0, and C_12_ < *B*_o_ < C_11_,^38^ except for Cs_3_AsI_6_. The comparatively lower elastic constant values for Rb_3_AsI_6_ relative to K_3_AsI_6_ indicate enhanced mechanical stability for Rb_3_AsI_6_. Conversely, the higher modulus values observed for K_3_AsI_6_, as reflected by the shear modulus GGG (GPa), bulk modulus BBB (GPa), and Young's modulus *Y* (GPa) presented in Table 3, account for its greater structural rigidity.

**Table 3:** Computed elastic possessions of the A_3_SbI_6_ (A = K, Rb, and Cs) compounds

Compounds	K_3_AsI_6_	Rb_3_AsI_6_	Cs_3_SbI_6_	Rb_3_SbI_6_
C_11_	43.21	32.00	12.45	30.34^a^
C_12_	2.25	4.70	9.39	3.26^a^
C_44_	19.74	11.58	−5.52	3.73^a^
Bulk modulus *B*_H_ [GPa]	15.90	13.80	Unstable, as it does not satisfy the Born Huang's stability criteria as C_44_ < 0	12.28^a^
Shear modulus *G*_H_ [GPa]	20.03	12.37		6.45^a^
Young's modulus *Y*_H_ [GPa]	42.33	28.57		16.48^a^
Poisson ratio *ν*_H_	0.05	0.15		0.28^a^
Pugh's ratio *B*/*G*	0.79	1.11		1.90^a^
Anisotropy *A*	0.001	0.01		0.28^a^
Debye temperature *θ*_D_ [K]	223.48	167.22		120.01^a^
Mean velocity *v*_m_ [m s^−1^]	2818.62	2152.36		1563.95^a^

a Ref. 22.

Table 3 shows that the bulk modulus obtained from structural optimization closely aligns with the values calculated using the elastic constant methods for A_3_SbI_6_ (A = K, Rb, and Cs). The distinction between ductile (*B*/*G* > 1.75) and brittle (*B*/*G* < 1.75) behavior in materials is defined by the critical threshold value of 1.75 in Pugh's ratio (*B*/*G*). The ductility of a material is further evaluated using Poisson's ratio (*ν*), where values exceeding 0.26 indicate ductile characteristics.^38^ The higher *B*/*G* and *ν* values for Rb_3_AsI_6_ confirm its enhanced ductility compared with K_3_AsI_6_. The sound velocity is calculated by taking the average of the transverse (*υ*_t_) and longitudinal (*υ*_L_) velocity components, calculated using the Navier equation of state (NEOS).^30,44,45^ However, K_3_AsI_6_ exhibits a higher average sound velocity than Rb_3_AsI_6_, which directly affects the Debye temperature, calculated based on the following equation:14
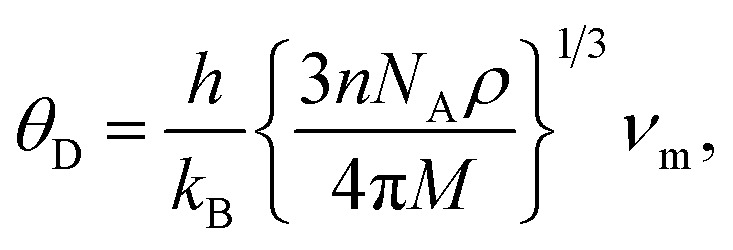
where *N*_A_ represents Avogadro's number, *k*_B_ denotes the Boltzmann constant, *ρ* is the material density, *M* denotes the molecular mass, and *v*_m_ denotes the mean sound velocity. The calculated Debye temperatures, listed in Table 3, demonstrate that K_3_AsI_6_ exhibits a higher value than Rb_3_AsI_6_. Since specific heat is directly related to the Debye temperature, these findings imply that K_3_AsI_6_ possesses a greater capacity to withstand heat generated from lattice vibrations.^29,46^ Additionally, the thermodynamic stability of these compounds tends to improve with increasing temperatures.

## 4 Phonon dispersion

**Fig. 7:**
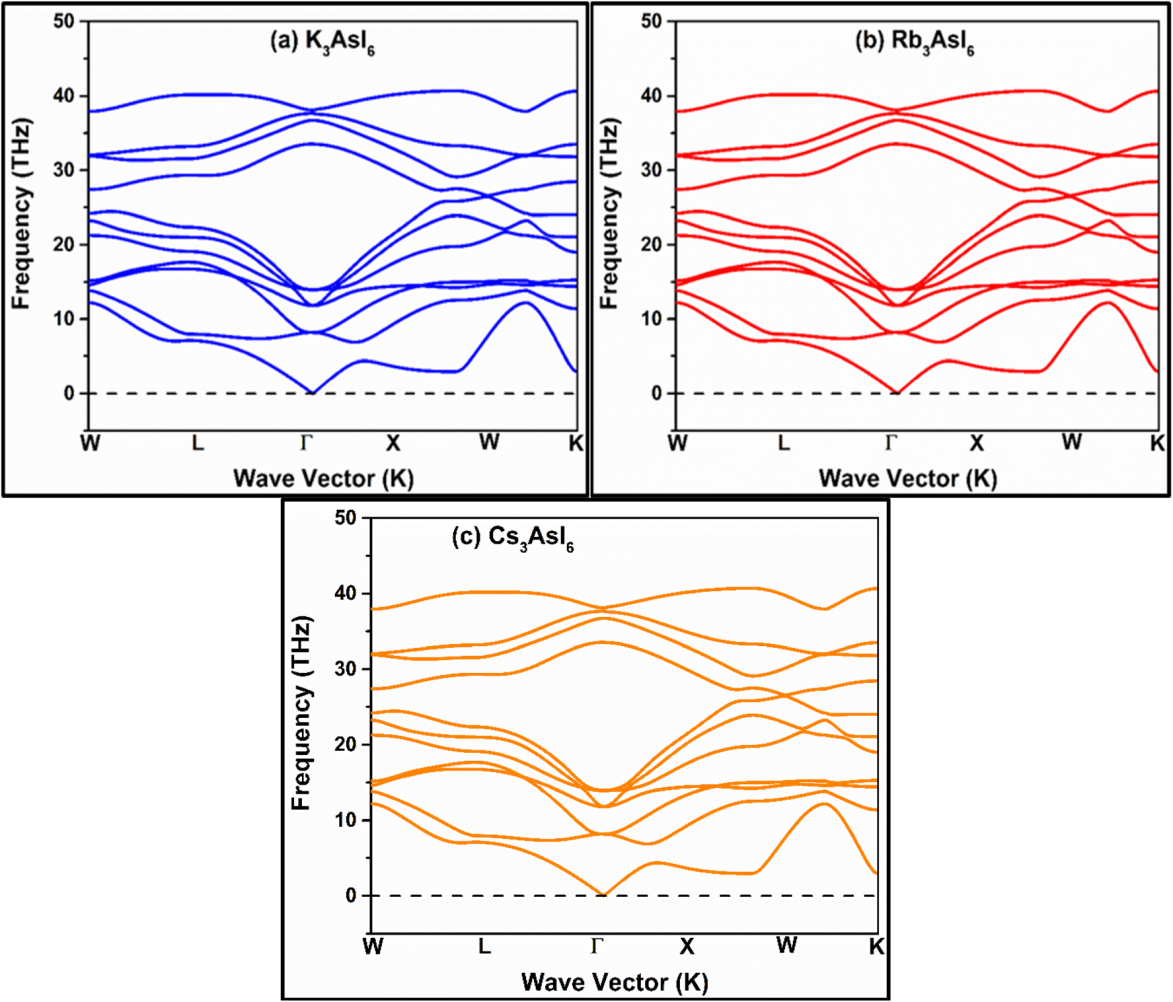
(a–c) Phonon dispersion plots of the A_3_AsI_6_ halide double perovskites, where A = K, Rb, and Cs, respectively.

The phonon dispersion spectra of K_3_AsI_6_, Rb_3_AsI_6_, and Cs_3_AsI_6_, computed *via* the linear-response formalism implemented in Materials Studio, distinctly reveal longitudinal optical–transverse optical (LO–TO) mode separation at the *Γ* point. This splitting originates from long-range coulombic forces inherent to polar crystalline lattices and is incorporated through non-analytic corrections to the dynamical matrix, which depend on the Born effective charge tensors and dielectric constants. Among these halide perovskites, K_3_AsI_6_ exhibits the largest LO–TO gap, signifying pronounced dipolar coupling, while Rb_3_AsI_6_ and Cs_3_AsI_6_ show progressive attenuation of this effect. The observed decline in splitting correlates with the systematic increase in ionic radius and atomic mass from K^+^ to Cs^+^, which mitigates the strength of long-range dipole–dipole interactions. Furthermore, the absence of imaginary frequencies across the entire Brillouin zone verifies the dynamical stability of all the three compounds (Fig. 7).

## 5 Conclusion

The structural, electronic, and optical characteristics of Pb-free mixed-halide double perovskites with the general formula of A_3_AsI_6_ (A = K, Rb, and Cs) were comprehensively explored using the full-potential linearized augmented plane wave (FP-LAPW) method within the framework of density functional theory (DFT). The computed electronic band structures indicate that these materials possess band gaps suitable for applications in light-emitting diodes (LEDs) and ultraviolet (UV) photodetectors. Optical properties were assessed using the Tran–Blaha modified Becke–Johnson generalized gradient approximation (TB-mBJ-GGA), both with and without spin–orbit coupling (SOC), revealing significant variations influenced by halide substitution. A systematic increase in lattice parameters is observed on moving from K to Cs. To date, no experimental or theoretical studies have been reported on these specific compounds. Among the compositions investigated, K_3_AsI_6_ demonstrates the most favorable characteristics for high-power radiation uncovering and UV optoelectronic applications owing to its superior optical conductivity and elevated absorption coefficient. Mechanical stability evaluations confirm that all phases, except Cs_3_AsI_6_, are mechanically robust. Additionally, A_3_AsI_6_ (A = K, Rb) exhibits brittle behavior, with Rb_3_AsI_6_ showing pronounced brittleness. These results provide deeper insight into lead-free double perovskites and offer a hypothetical root for future untried studies and the potential design of advanced optoelectronic devices.

## Conflicts of interest

There are no conflicts to declare.

## Data Availability

The data supporting the findings of this study are available from the corresponding author upon reasonable request. All computational input files, output data, and analysis scripts related to the first-principles calculations of the As-based mixed halide double perovskites A_3_AsI_6_ (A = K, Rb, and Cs), including the structural optimizations, electronic band structures, density of states, phonon dispersions, and optical property simulations, were archived and can be provided for academic and non-commercial research purposes.
